# Comparison of unattended, attended and routine clinic systolic blood pressure measurements and determinants of blood pressure difference between attended and unattended BP

**DOI:** 10.1038/s41371-026-01162-5

**Published:** 2026-05-27

**Authors:** Fraser Todd, Joseph Rushton, Emily Hayashi, Hannah Brown, Cynthia Nguyen, Emmeline Lai, Emily Schembri, Gary Yip, Mahesan Anpalahan

**Affiliations:** 1 Department of General Medicine, Eastern Health, Melbourne, VIC Australia; 2 Department of Medicine, Monash University, Melbourne, VIC Australia

**Keywords:** Diagnosis, Hypertension

## Abstract

The study compared unattended(uAOBP), attended(aAOBP) and routine clinic systolic blood pressures (SBP) and investigated the role of BP measurement sequence and aAOBP threshold on SBP difference between aAOBP and uAOBP. Eligible patients were randomized to the order of aAOBP and uAOBP measurements during a single clinic visit. aAOBP and uAOBP measurements were obtained under standardised conditions using an Omron HEM907 BP-monitor. Routine clinic BP(CBP) from the same visit was also obtained. 85 patients (mean age 69.9 ± 16.4 years, 52.9% males) underwent aAOBP and uAOBP measurements. CBP was available in 78. uAOBP was significantly lower than both aAOBP (mean difference 4.12 ± SD7.91 mmHg, *P* < 0.001) and CBP (mean difference 6.67 ± SD6.67 mmHg, *P* < 0.001). When comparing randomised groups, mean BP difference was significant in only those who underwent aAOBP measurement first (6.30 + 7.29 mmHg; *P* < 0.001), but not in those who underwent uAOBP measurement first (1.88 + 7.98 mmHg; *P* = 0.134). In subgroup analysis by aAOBP ≥130 mmHg(vs.<130 mmHg), the mean BP difference was significant in only those with an aAOBPs ≥130 mmHg (6.13 ± 0.07 mmHg, *P* < 001), but not in those with an aAOBPs <130 mmHg (1.63 + 7.05 mmHg, *P* = 0.162). Correlation and Bland-Altman analyses revealed closer correlation between uAOBP and aAOBP(r = 0.925, *P* < 0.001), with lower bias (4.12 mmHg) and narrower 95% limits of agreement (LoA) (−11.38, 19.62 mmHg), compared to clinic SBP (r = 0.784, *P* < 0.001; Bias: 6.67 mmHg; LoA:−20.67, 34.01 mmHg. An aAOBP of 140 mmHg corresponded to an uAOBP of 135.3 mmHg. Both CBP and aAOBP significantly overestimate uAOBP. BP differences are partly attributable to the sequence of BP measurement and the level of aAOBP.

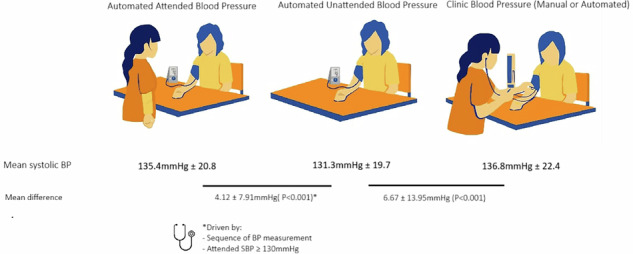

## Introduction

Accurate measurement of blood pressure for the diagnosis of hypertension and treatment monitoring remains a challenge. Blood pressure in routine practice is usually measured using an automated oscillometric device with a clinician present, and this may cause falsely elevated blood pressure due to the white coat effect [1]. In 2015, the Systolic Blood Pressure Intervention Trial (SPRINT) brought the concept of unattended automated blood pressure measurement (uAOBP) to the forefront of blood pressure assessment [2]. Automation mitigates the risk of observer bias and readings taken with the patient alone reduces the risk of white coat effect [3].

Despite these advantages, the suitability of uAOBP as the standard method for routine office BP assessment remains controversial, with recommendations in guidelines ranging from a clear preference [4] to admonishment [5]. This may be because of a lack of strong evidence for predicting outcomes for uAOBP compared to attended automated office BP (aAOBP) [6–8]. Furthermore, studies comparing uAOBP and aAOBP techniques have produced mixed results in blood pressure readings [3, 9, 10]. Thus, we aimed to compare unattended, attended and routine office or clinic systolic blood pressures in patients attending outpatient medical clinics. Additionally, we investigated the potential modifying effect of BP measurement sequence and the attended BP threshold <130 mmHg (vs. ≥130) on the mean BP difference between aAOBP and uAOBP measurements. We also investigated the potential associations of demographic and clinical variables with the mean BP difference between attended and unattended BPs.

## Materials and methods

### Design, setting and participants

This was a prospective, observational study conducted at Eastern Health, a tertiary teaching hospital network in Melbourne, Australia, where patients were randomly assigned to the measurement sequence of aAOBP and uAOBP. Eligible participants included all patients aged 18 or over with a documented history of hypertension and/or cardiovascular comorbidities attending medical outpatient clinics at Eastern Health. Data collection occurred between November 2023 and February 2025. Patients were approached during their clinic visit and provided with written information about the study. After obtaining informed verbal consent participants were randomised in a 1:1 ratio to one of two sequences: (1) uAOBP followed by aAOBP, or (2) aAOBP followed by uAOBP.

### Blood pressure measurement

Both aAOBP and uAOBP measurements were performed using the Omron HEM-907 automated BP monitor. Measurements were taken using an appropriately sized cuff, with the patient seated comfortably and resting for five minutes prior to the first reading, in accordance with current blood pressure guidelines [5]. For the uAOBP measurement, the device was programmed to take three BP measurements, the first after a five-minute rest followed by two additional readings at one-minute intervals. After activating the device, the investigator left the room and only returned after the three readings had been complete. For the aAOBP measurement, the investigator remained present both during the five-minute rest and measurement periods and manually activated each reading at one-minute intervals. The mean value of the three blood pressure readings was used for analysis. During the aAOBP measurement no interaction or conversation was permitted between the patient and investigator. The patient was also not allowed to use cell phone. The investigator remained in the room but did not engage with the patient and quietly worked on the computer, collecting relevant study data from the electronic medical records.

Routine clinic blood pressures taken by the treating clinician in the same visit were obtained retrospectively from medical records. Routine clinic blood pressures were obtained using either manual auscultatory or automated monitors at the discretion of the clinic physician and depending on device availability. The clinics were staffed by consultant physicians and trainee physicians working under supervision. Investigators were not present during routine clinic BP measurements and deliberately minimised any interactions with the clinic staff to avoid any potential influence of the study on usual clinical practice. As investigators were not present during clinic BP measurements, it is difficult to comment on the specific conditions under which routine clinic BPs were obtained, but they were presumably measured under a mixture of standardised, semi-standardised and non-standardised conditions.

Relevant demographic and clinical data were collected from electronic medical records, and included age, sex, weight, height, comorbidities and current medications. The study was approved by the Eastern Health Human Research Ethics Committee (approval No LR23-056-101611) and was conducted in accordance with the ethical guidelines of the Declaration of Helsinki.

### Statistical analysis

A sample size of 70 (35 in each arm) was estimated to give a statistical power of at least 80% with a two-sided alpha significance level of 0.05, calculated using a paired t test assuming a standard deviation of difference of 8.6 mmHg [11] to detect a difference of 3 mmHg in mean blood pressure difference between attended and unattended blood pressures. A permuted block randomisation technique was used to allocate patients to the two blood pressure measurement sequences.

Patient characteristics were summarised using mean (SD) for continuous variables (after assessing normality using Shapiro-Wilk test) and frequency (proportion) for categorical variables. Attended, unattended and clinic blood pressure results were summarised using mean (SD) following assessment of normality and of homogeneity of variance using Levene’s test. Within- patient differences between BP measurements were calculated for each patient and summarised using mean (SD). Repeated measures ANOVA, followed by post hoc analysis using Tukey’s method, was used to assess differences in BP measurements across the three settings. Greenhouse–Geisser corrections were examined for potential violations of the sphericity assumption. Students T test was used to determine differences between attended and unattended BP and in those who had attended BP taken first, compared to those who had unattended BP taken first. Bland-Altman Plots were used to assess agreement and biases between the three settings. Pearson correlation was used to assess correlations between attended, unattended and clinic BP measurements, and displayed using scatter plots. Subgroup analyses were preformed to assess differences between patients with attended systolic BP of less than 130 mmHg, and equal to, or above 130 mmHg. Linear regression analysis, with assessment of model assumptions, was performed to determine if particular variables were associated with an increased mean difference between attended and unattended SBP. The model was also used to establish the corresponding unattended systolic BP value for the attended BP value of 140 mmHg, the systolic BP threshold that define hypertension by most BP guidelines [5]. A two-sided P value of < 0.05 was considered statistically significant. Statistical analyses were performed using STATA v.18.

## Results

In total, 85 patients underwent aAOBP and uAOBP measurements. The mean age was 69.9 ± 16.4 years, and 45 (52.9%) were males. Demographic, and clinical characteristics of the sample are summarised in Table 1. Seven patients did not have a contemporaneous routine clinic blood pressure measurement available for analysis and were not included in threeway measurement comparisons. Mean aAOBP, uAOBP and routine clinic systolic blood pressures (CBP) were 135.4 mmHg ± 20.8, 131.3 mmHg ± 19.7 and 136.8 mmHg ± 22.4 respectively. All three settings of BP were normally distributed and demonstrated homogeneity of variance (Levene’s test, p = 0.260). uAOBP was significantly lower than both aAOBP (mean difference 4.12 mmHg, *P* < 0.001) and clinic (mean difference 6.6 mmHg, *P* < 0.001) systolic BP readings. However, when comparing randomised groups, mean BP difference between aAOBP and uAOBP was significant in only those who underwent aAOBP measurement first (6.30 ± 7.29 mmHg, *P* < 0.001), and not in those who underwent uAOBP measurement first (1.88 ± 7.98 mmHg, *P* = 0.134) (Table 2).

**Table 1:** Summary of participant characteristics.

Characteristics	Total population
**N**	85
**Age, years**, mean (SD)	69.9 (16.4)
**Weight**, kg, mean (SD)	87.9 (27.5)
**Height**, cm, mean (SD)	167.1 (10.6)
**BMI**, kg/m, mean(SD)	31.4 (8.6)
**Sex**, n(%)	
Female	40 (47.1)
Male	45 (52.9)
**Comorbidities**, n(%)	
HTN	66 (77.7%)
IHD	19 (22.4%)
DM	32 (37.7%)
CKD	35 (41.2%)
CVA/TIA	9 (10.6%)
Smoking (Current or historical)	28 (32.9%)
Hyperlipidaemia	52 (61.2%)
COPD	9 (10.6%)
CCF	24 (28.2%)
PVD	14 (16.5%)
**Medications**, n(%)	
ACEI/ARB	52 (61.2%)
BB	34 (40.0%)
CCB	32 (37.7%)
Thiazide	9 (10.6%)
MRA	23 (27.1%)
Furosemide	31 (36.5%)
Other	9 (10.6%)
**Number of blood pressure medications**, median (IQR)	2 (1–3)

*HTN*, hypertension; *IHD*, ischaemic heart disease; *DM*, diabetes mellitus; *CKD*, chronic kidney disease, *CVA/TIA*, cerebrovascular disease/transient ischaemic attack; *COPD*, chronic obstructive pulmonary disease; *CCF*, congestive cardiac failure; *PVD*, peripheral vascular disease; *ACEI/ARB*, angiotensin-converting enzyme inhibitor/angiotensinogen II receptor blocker; *BB*’, beta blocker; *CCB*, calcium channel blocker; *MRA*, mineralocorticoid receptor antagonist.

**Table 2:** Effect of sequence of BP measurement, and attended or routine clinic BP (<130 vs. ≥130mm Hg) on mean differences in SBP, in mmHg.

	Mean BP (SD)	Mean Difference (SD)	*P* Value
Difference between sequences			
Attended BP first (*n* = 43)			
Attended	141.42 (21.29)	6.30 (7.29)	<0.001
Unattended	135.12 (19.75)		
Unattended BP first (*n* = 42)			
Attended	127.43 (19.02)	1.88 (7.98)	0.134
Unattended	129.31 (18.56)		
Difference between </≥ 130 Attended SBP			
Attended SBP < 130 mmHg (*n* = 38)			
Attended	117.55 (9.71)	1.63 (7.05)	0.162
Unattended	115.92 (10.97)		
Attended SBP ≥ 130 mmHg (*n* = 47)			
Attended	149.89 (15.33)	6.13 (8.07)	<0.001
Unattended	143.77 (15.97)		
Difference between </≥ 130 Clinic SBP			
Clinic SBP < 130 mmHg (*n* = 34)			
Attended	120.15 (13.64)	2.71 (7.03)	0.032
Unattended	117.44 (14.55)		
Clinic SBP ≥ 130 mmHg (*n* = 44)			
Attended	144.86 (18.03)	4.91 (8.72)	<0.001
Unattended	139.95 (16.03)		

*SBP*, systolic blood pressure; *SD*, standard deviation.

In the prespecified subgroup analysis by an aAOBP threshold ≥130 mmHg vs. < 130 mmHg, the mean BP difference was significant in only those with an aAOBP > 130 mmHg (6.13 mmHg ±8.07, *P* < 001) and not in those with aAOBPs < 130 mmHg (1.63 mmHg ± 7.05, *P* = 0.162) (Table 2). Subgroup analysis by routine clinic B*P* ≥ 130 mmHg vs. < 130 mmHg revealed a similar pattern in that the mean BP difference between aAOBP and uAOBP was greater (4.91 mmHg + 8.72, *P* < 0.001) in those with a systolic CB*P* ≥ 130 compared to those with a clinic SB*P* < 130 mmHg (2.71 mmHg + 7.03, *P* = 0.032), although mean BP differences were significant in both groups (Table 2).

Post-hoc analysis of the sequence of BP measurement, viz. aAOBP first vs. uAOBP first, when stratified by attended or clinic systolic B*P* < 130 mmHg vs. ≥130 mmHg revealed that the mean BP difference was higher when aAOBP was performed first compared to when uAOBP was performed first; however, the mean BP difference was significant only in those with an attended or routine clinic B*P* ≥ 130 mmHg, and not in those with a B*P* < 130 mmHg (Supplementary Table 1).

**Fig. 1:**
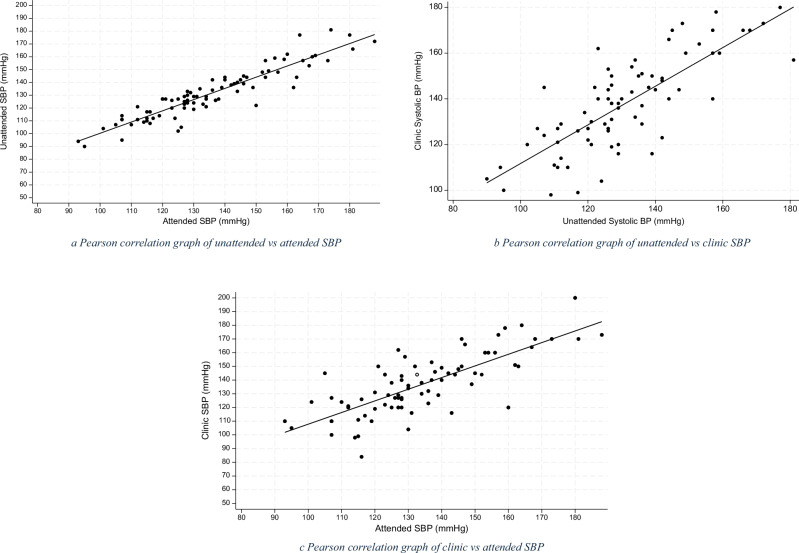
Pearson correlation graphs between unattended, attended and clinic systolic blood pressures.

**Fig. 2:**
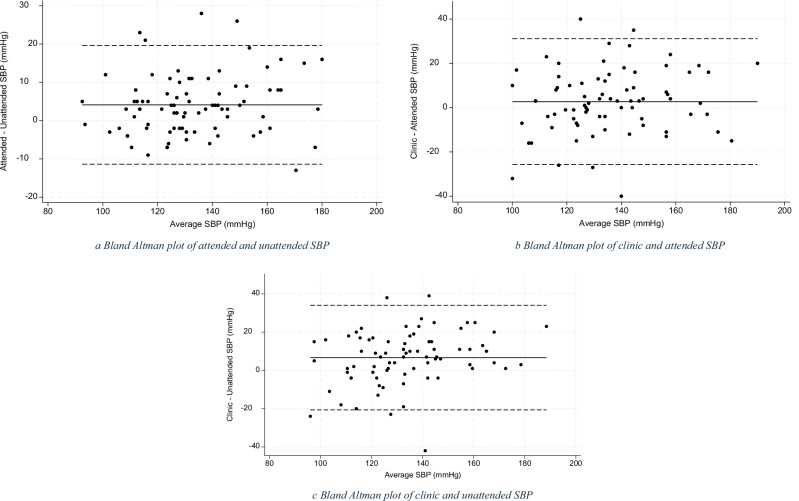
Bland Altman Plots between unattended, attended and clinic systolic blood pressures.

Scatter plot and correlation coefficient for systolic BP between uAOBP and aAOBP revealed closer correlation (r = 0.925, p < 0.001) compared to that between uAOBP and CBP (r = 0.784, *P* < 0.001) (Table 3, Fig. 1a–c). Similarly, Bland-Altman analyses between uAOBP and aAOBP revealed lower bias (4.12 mmHg) and narrower 95% limits of agreement (LoA) (−11.38, 19.62 mmHg) compared to that between uAOBP and clinic SBP (r = 0.784, *P* < 0.001; Bias: 6.67 mmHg; LoA: −20.67, 34.01 mmHg (Table 3, Fig. 2a–c). Attended SBP of 140 mmHg corresponded to an unattended SBP of 135.3 mmHg in linear regression.

**Table 3:** Summary of systolic blood pressure (SBP) comparisons.

	N	Mean difference (Bias)	SD of Differences	P value	Limits of Agreement	Pearson’s Correlation
Attended Vs Unattended	85	4.12	7.91	<0.001	−11.38, 19.62	r = 0.925, P < 0.001
Clinic Vs Attended	78	2.72	14.51	0.137^	−25.71, 31.15	r = 0.773; P < 0.001
Clinic Vs Unattended	78	6.67	13.95	<0.001^	−20.67, 34.01	r = 0.784, P < 0.001

Pairwise comparisons of SBP readings, in mmHg ^Tukey adjusted P value reported.

Linear regression analysis showed no significant association of mean BP difference between aAOBP and uAOBP with any of the demographic (age, gender, weight, BMI) or comorbid variables (hypertension, ischaemic heart disease, diabetes mellitus, chronic kidney disease, cerebrovascular events, heart failure, peripheral vascular disease, chronic obstructive pulmonary disease, smoking, hyperlipidaemia, or blood pressure medication use (results not shown).

## Discussion

In this randomised real-world trial conducted in a population of medical outpatients, we have demonstrated that unattended systolic blood pressure is significantly lower than both attended and routine clinic blood pressures. The difference is both statistically and clinically significant. The difference in systolic blood pressure (SBP) between aAOBP and uAOBP appears to be primarily driven by those who had aAOBP measurement performed first and those with an attended or clinic systolic B*P* ≥ 130 mmHg. Our study also demonstrates that a systolic aAOBP of 140 mmHg corresponded to an uAOBP of approximately 135 mmHg.

Although previous studies have compared attended, unattended, and clinic blood pressure (BP) measurements, only a limited number have employed a methodology as robust as that of the present study. In particular, patients were randomly allocated to the BP measurement sequence to minimise any order effect. Furthermore, subgroup analyses were conducted within each sequence to detect any residual effect of measurement order. This represents an important methodological advance over previous studies and strengthens the validity of our findings.

Previous studies have yielded mixed results. While some studies [3, 8, 12–15] and meta-analyses [10, 16], consistent with the finding of the present study, have shown that unattended SBP is significantly lower than attended and clinic SBPs, others have produced contrasting results [9, 17–19]. Even among studies demonstrating a significant difference, the magnitude of the mean BP difference between aAOBP and uAOBP has varied considerably [1, 6, 10, 12]. Such heterogeneity may be attributable to methodological limitations, including failure to randomise patients to BP measurement sequence. In addition, differing population characteristics, study settings and study protocols may have also contributed. As far as population characteristics are concerned, as observed by Myers [20], lower or within target baseline blood pressure has been associated with a narrower [3] or no difference [18] between attended and unattended AOBP measurements. In this context, the mean BP difference observed in our study lies between the 2.7 mmHg reported by Keely et al. [3] and the higher mean difference of 8.6 mmHg reported by Paini et al. [14], and is broadly consistent with the baseline attended BP levels reported in these study cohorts. With respect to study settings, studies undertaken in familiar medical environments to patients have been shown to minimise BP difference between attended and unattended BP [9]. Furthermore, as evidenced by systematic reviews and meta-analyses [10, 16, 21] variations in study protocols too have been a major source of heterogeneity and include antecedent rest period [22, 23], number of BP measurements obtained [24], devices used [25] and clinician type [26].

Although the modulatory effect of the sequence of BP measurement on mean BP difference between aAOBH and uAOBP has been recognised in the current literature [10], this issue has been explicitly investigated by only a few studies. In the meta- analysis of 12 studies, Andreadis et al. [10] observed that the mean BP difference between aAOBP and uAOBP was narrower or insignificant in studies that randomised patients to BP measurement sequence as opposed to studies where attended BP was performed first. Of note, in almost all studies where attended BP was performed first, the mean BP difference was found to be statistically significant. Although some studies have randomised measurement sequence to mitigate the order effect [3, 8, 9], to our knowledge, apart from the present study, only one other study, the study by Fanelli et al., reported the results of subgroup analysis of the sequence of BP measurement [11]. Consistent with our study, the findings of Fanelli et al. [11] also suggested that the sequence of BP measurement is an important determinant of BP difference between aAOBP and uAOBP. Keeley et al. [3] reported no order effect to the sequence of BP measurement, but the study did not provide the results of subgroup analysis. Their findings are at variance with ours and that of Fanelli et al. but this may be due to the low baseline BP of the sample in their study [20]. A narrower or no difference in mean BP between aAOBP and uAOBP in those who undergo aAOBP measurement second may be explained by a longer rest time [21, 27] and a period of habituation to repeated BP measurements when one undergoes aAOBP measurement second, thus potentially dampening the white coat effect.

The BP threshold of <130 vs. ≥130 mmHg was chosen for subgroup analysis because it defines hypertension by the American hypertension guidelines [28]. Furthermore, previous studies have indicated that the prevalence white coat effect is low at BP levels below 130 mmHg [29]. In subgroup analysis, we demonstrated that patients with an aAOBP or routine clinic SB*P* ≥ 130 had a significantly greater difference between aAOBP and uAOBP readings compared to those with an aAOBP or routine clinic SB*P* < 130. This finding further corroborates existing reports indicating that white coat effect progressively attenuates at lower clinic or attended BP levels and disappears at normotensive levels below 120–130 mmHg [17, 29–31]. Myers et al. in their review concluded that white coat effect is not a concern in patients with a systolic B*P* < 130 mmHg [29], The results of the ONTARGET trial [30] demonstrated that the 24-hour systolic BP was ≈ 14 mm Hg lower than clinic systolic BP at baseline, whereas during treatment the two values became progressively closer as clinic systolic BP was more tightly controlled and superimposable when clinic systolic BP was <120 mm Hg. A similar observation was made by the authors of PAMELA study in an unbiased sample of general population [31]. In keeping with these ambulatory blood pressure (ABP) studies, studies and meta-analyses comparing aAOBP and uAOBP measurements also confirmed a trend toward narrower difference between aAOBP and uAOBP at a lower attended blood pressure [14, 17, 29]. In this context, it is worth noting that several studies have shown that AOBP readings, both attended and unattended, may be lower than day-time ambulatory BP readings at the lower end of the normotensive range [29]. However, exploring this observation is beyond the scope of the present study. In addition, studies have suggested that the technique of BP measurement whether unattended, attended, ambulatory or manual makes little difference in the normotensive BP levels (<130 mmHg) [29]. It is also worth noting that studies have indicated that the antecedent rest period is less relevant in those with an attended B*P* < 130 mmHg [23]. Taken together, these findings would suggest that BP measurement techniques may not be particularly relevant at BP levels in the normotensive range.

We observed that an attended SBP of 140 mmHg corresponded to an unattended SBP of approximately 135 mmHg, the BP threshold that defines hypertension by both day-time ambulatory and home BP (HBP) measurements. This is in keeping with previous studies that have shown a close correlation between unattended and ambulatory blood pressures [16, 32]. Roerecke et al. in a meta-analysis of 19 studies demonstrated that the mean systolic uAOBP was only 0.3 mmHg different from the mean awake systolic ABP [16]. Myers et al. in sample of 514 untreated patients referred for ambulatory BP monitoring, showed that the mean adjusted uAOBP values of 130 and 135 mmHg systole corresponded to mean awake systolic ABP values of 132.1 and 134.4 mmHg, respectively [32] These observations would suggest that uAOBP could be an alternative to day time ABP or HBP monitoring to diagnose white coat HT or white coat effect and if unattended BP was to be adopted for routine clinical use for measuring clinic BP, a SBP threshold of >135 mmHg and not ≥140 mmHg systole should be used to define hypertension or suboptimal BP control and to diagnose other phenotypes of hypertension such as masked hypertension [6, 17].

Studies have found female gender [14], older age, duration of hypertension, family history of hypertension, obesity, non-smoking status and treatment with anti-hypertensives to be independently associated with white coat hypertension or white coat effect [33]. Therefore, it would be reasonable to hypothesise that these factors may have a significant association with BP difference between aAOBP and uAOBP. However, we found no significant association between any demographic or clinical variables and the difference between aAOBP and uAOBP readings. In agreement with our results, Kollias et al. [17] found no association between age or gender and mean BP difference in their meta-analysis. In contrast, Paini et al., reported a significant association with older age and female gender [14]. Triantafyllidi et al. [34] on the other hand found no association with age, smoking, obesity or control of hypertension in hypertensive patients, although older age, non-smoking and non-diabetic status were found to have a significant association with mean BP difference in normotensive individuals. As can be seen, only limited studies have commented on these associations and the findings remain inconsistent to make any firm conclusions.

### Strengths and limitations

The results of this study should be interpreted within the context of potential strengths and weaknesses. The study did not incorporate out of office BP measurements (neither ambulatory (ABPM) nor home blood pressure monitoring (HBPM)); therefore, a direct comparison between uAOBP and daytime ABP or HBP readings was not possible. Although the study was powered to detect a 3 mmHg mean difference between aAOBP and uAOBP in the overall sample, it lacked sufficient statistical power to detect a similar difference in subgroup analyses, such as those stratified by BP measurement sequence or by an attended or routine clinic BP threshold of <130 mmHg vs. ≥130 mmHg. Our findings strongly suggest that the sequence of BP measurement modulates mean BP difference. However, to arrive at firmer conclusions regarding the modifying effect of measurement sequence, further evaluation would be required in a crossover study with reversal of BP measurement sequence within the same individuals under controlled conditions. Finally, while these results provide valuable insights into unattended BP measurement in a cohort of patients with hypertension or cardiovascular comorbidities, they should be interpreted with caution when extrapolated to the diagnosis of hypertension in treatment-naïve patients.

Despite these limitations, the study confirms that both attended and clinic systolic blood pressures significantly overestimate unattended systolic blood pressure, a finding likely to have important clinical implications. Although aAOBP readings may approximate uAOBP when measured after uAOBP, implementing such a BP measurement sequence in a real-world setting is neither practical nor time efficient; therefore, the modulatory effect of measurement sequence does not diminish the clinical relevance of uAOBP. Furthermore, studies have demonstrated that uAOBP measurement protocol can be significantly shortened without compromising the accuracy of BP measurement, thereby improving the feasibility and clinical applicability of this technique in a busy clinic environment [22]. The study also provides further evidence for a lower prevalence of white coat hypertension or white coat effect below an aAOBP or routine clinic BP level of 130 mmHg - in other words in those with SBP readings within target levels. In addition, the findings strengthen the evidence supporting a closer correlation between uAOBP and mean daytime ABP or HBP readings.

Overall, the findings of this study suggest that patients, particularly those with attended or clinic systolic BP $\ge$130 mmHg, would benefit from unattended AOBP measurement, as it is likely to improve the accuracy of BP measurement. The findings further strengthen the existing evidence base on unattended BP measurement, particularly in light of the methodological advances incorporated into the present study, and underscore the rationale for broader implementation of uAOBP in routine clinical practice. Although the present study did not assess the feasibility of implementing uAOBP in routine clinical settings, as discussed previously, other studies have demonstrated its practicality in real-world settings. Outcome-based and interventional studies directly comparing aAOBP, uAOBP and ABP are needed to further strengthen and validate the evidence supporting the routine clinical use of uAOBP.

## SUMMARY Table

### What is known about topic

1. Studies comparing attended and unattended BP measurements have produced mixed results.
2. Determinants of BP difference between attended and unattended BP measurements are poorly characterised.

### What this study adds

1. Both routine clinic BP and attended automated office BP(aAOBP) significantly overestimate unattended automated office BP (uAOBP).
2. BP differences between aAOBP and uAOBP are at least in part due to factors associated with the sequence of BP measurement and the level of aAOBP or routine clinic BP.
3. The prevalence of white coat hypertension or white coat effect attenuates when aAOBP or clinic BP is <130mmgHg.

## Supplementary information

Supplementary Table 1

## Data Availability

The datasets generated and/or analysed during the current study are not publicly available, but reasonable requests for deidentified data will be considered for sharing after obtaining approval from the institutional ethics committee.

## References

[1] Gorostidi M, Vinyoles E, Banegas JR, de la Sierra A. Prevalence of white-coat and masked hypertension in national and international registries. Hypertens Res. 2015;38:1–7. 25319601 10.1038/hr.2014.149

[2] SPRINT Research Group, Wright JT Jr, Williamson JD, Whelton PK, Snyder JK, Sink KM, Rocco MV, et al. A randomized trial of intensive versus standard blood-pressure control. N Engl J Med. 2015;373:2103–16. Epub 2015 Nov 9. Erratum in: N Engl J Med. 2017 Dec 21;377(25):2506. PMID: 26551272 26551272 10.1056/NEJMoa1511939 PMC4689591

[3] Keeley EC, Villanueva M, Chen YE, Gong Y, Handberg EM, Smith SM, et al. Attended vs unattended systolic blood pressure measurement: A randomized comparison in patients with cardiovascular disease. J Clin Hypertens (Greenwich). 2020;22:1987–92. 32951360 10.1111/jch.14037 PMC8029864

[4] Rabi DM, McBrien KA, Sapir-Pichhadze R, Nakhla M, Ahmed SB, Dumanski SM, et al. Hypertension Canada’s 2020 comprehensive guidelines for the prevention, diagnosis, risk assessment, and treatment of hypertension in adults and children. Can J Cardiol. 2020;36:596–624. 32389335 10.1016/j.cjca.2020.02.086

[5] McEvoy JW, McCarthy CP, Bruno RM, Brouwers S, Canavan MD, Ceconi C, et al. 2024 ESC Guidelines for the management of elevated blood pressure and hypertension. Eur Heart J. 2024;45:3912–4018. Erratum in: Eur Heart J. 2025 Apr 7;46(14):1300 PMID: 39210715 39210715 10.1093/eurheartj/ehae178

[6] Myers MG, Kaczorowski J, Paterson JM, Dolovich L, Tu K. Thresholds for diagnosing hypertension based on automated office blood pressure measurements and cardiovascular risk. Hypertension. 2015;66:489–95. 26269653 10.1161/HYPERTENSIONAHA.115.05782

[7] Myers MG, Kaczorowski J, Dolovich L, Tu K, Paterson JM. Cardiovascular risk in hypertension in relation to achieved blood pressure using automated office blood pressure measurement. Hypertension. 2016;68:866–72. 27528062 10.1161/HYPERTENSIONAHA.116.07721

[8] Salvetti M, Paini A, Aggiusti C, Bertacchini F, Stassaldi D, Capellini S, et al. Unattended versus attended blood pressure measurement. Hypertension. 2019;73:736–42. 30686088 10.1161/HYPERTENSIONAHA.118.12187

[9] Bauer F, Seibert FS, Rohn B, Bauer KAR, Rolshoven E, Babel N, et al. Attended versus unattended blood pressure measurement in a real life setting. Hypertension. 2018;71:243–9. 29255074 10.1161/HYPERTENSIONAHA.117.10026

[10] Andreadis EA, Thomopoulos C, Geladari CV, Papademetriou V. Attended versus unattended automated office blood pressure: a systematic review and meta-analysis. High Blood Press Cardiovasc Prev. 2019;26:293–303. 31290085 10.1007/s40292-019-00329-1

[11] Fanelli E, Di Monaco S, Pappaccogli M, Eula E, Fasano C, Bertello C, et al. Comparison of nurse attended and unattended automated office blood pressure with conventional measurement techniques in clinical practice. J Hum Hypertens. 2022;36:833–8. 34285354 10.1038/s41371-021-00575-8

[12] Bertram S, Bauer F, Shadi R, Seidel M, Doevelaar A, Seibert F, et al. Prevalence of masked hypertension in attended versus unattended office blood pressure measurement. J Clin Hypertens (Greenwich). 2024;26:615–23. 38751130 10.1111/jch.14798 PMC11180678

[13] Bartoloni E, Angeli F, Marcucci E, Perricone C, Cafaro G, Riccini C, et al. Unattended compared to traditional blood pressure measurement in patients with rheumatoid arthritis: a randomised cross-over study. Ann Med. 2021;53:2050–9. 34751628 10.1080/07853890.2021.1999493 PMC8583925

[14] Paini A, Bertacchini F, Stassaldi D, Aggiusti C, Maruelli G, Arnoldi C, et al. Unattended versus attended blood pressure measurement: Mean values and determinants of the difference. Int J Cardiol. 2019;274:305–10. 29945805 10.1016/j.ijcard.2018.06.056

[15] Paiva AMG, Mota-Gomes MA, Feitosa ADM, Azevedo TCP, Amorim NW, Mion D Jr, et al. Differences in the diagnosis of high blood pressure using unattended and attended automated office blood pressure. J Hum Hypertens. 2022;36:370–2. 34404899 10.1038/s41371-021-00593-6

[16] Roerecke M, Kaczorowski J, Myers MG. Comparing automated office blood pressure readings with other methods of blood pressure measurement for identifying patients with possible hypertension: a systematic review and meta-analysis. JAMA Intern Med. 2019;179:351–62. 30715088 10.1001/jamainternmed.2018.6551 PMC6439707

[17] Kollias A, Stambolliu E, Kyriakoulis KG, Gravvani A, Stergiou GS. Unattended versus attended automated office blood pressure: Systematic review and meta-analysis of studies using the same methodology for both methods. J Clin Hypertens (Greenwich). 2019;21:148–55. 30585383 10.1111/jch.13462 PMC8030301

[18] Andreadis EA, Geladari CV, Angelopoulos ET, Savva FS, Georgantoni AI, Parademetriou V. Attended and unattended automated office blood pressure measurements have better agreement with ambulatory monitoring than conventional office readings. J Am Heart Assoc. 2018;7:e008994. 29627767 10.1161/JAHA.118.008994 PMC6015428

[19] Papademetriou V, Tsioufis C, Chung A, Geledari C, Andreadis EA. Unobserved automated office BP is similar to other clinic BP measurements: A prospective randomized study. J Clin Hypertens (Greenwich). 2018;20:1411–6. 30272388 10.1111/jch.13371 PMC8030999

[20] Myers MG. Attended automated office blood pressure re-visited. J Clin Hypertens (Greenwich). 2020;22:1993–4. 32986916 10.1111/jch.14031 PMC8029907

[21] Myers MG, Godwin M, Dawes M, Kiss A, Tobe SW, Kaczorowski J. Conventional versus automated measurement of blood pressure in the office (CAMBO) trial. Fam Pract. 2012;29:376–82. 22117083 10.1093/fampra/cmr113

[22] Lynn-Green EE, Cluett JL, Turkson-Ocran RN, Mukamal KJ, Li JX, Jaraschek SP. Clinical impact of 3- Vs. 5-minute delay and 30- Vs. 60-second intervals on unattended automated office blood pressure measurements. Am J Hypertens. 2025;ume 38:168–77. 10.1093/ajh/hpae135 PMC11833244 39387134

[23] Colella TJF, Tahsinul A, Gatto H, Oh P, Myers MG. Antecedent rest may not be necessary for automated office blood pressure at lower treatment targets. J Clin Hypertens (Greenwich). 2018;20:1160–4. Epub ahead of print. PMID: 29900674. 29900674 10.1111/jch.13319 PMC8030766

[24] Desbiens LC, Nadeau-Fredette AC, Madore F, Agharazii M, Goupil R. Impact of successive office blood pressure measurements during a single visit on cardiovascular risk prediction: analysis of CARTaGENE. Hypertension. 2023;80:2209–17. 37615094 10.1161/HYPERTENSIONAHA.123.21510

[25] Rinfret F, Cloutier L, Wistaf R, Birnbaum LM, Cheong NN, Laskine M, et al. Comparison of different automated office blood pressure measurement devices: evidence of nonequivalence and clinical implications. Can J Cardiol. 2017;33:1639–44. 29173603 10.1016/j.cjca.2017.09.011

[26] Clark CE, Horvath IA, Taylor RS, Campbell JL. Doctors record higher blood pressures than nurses: systematic review and meta-analysis. Br J Gen Pract. 2014;64:e223–32. 24686887 10.3399/bjgp14X677851 PMC3964448

[27] Nikolic SB, Abhayaratna WP, Leano R, Stowasser M, Sharman JE. Waiting a few extra minutes before measuring blood pressure has potentially important clinical and research ramifications. J Hum Hypertens. 2014;28:56–61. 23719215 10.1038/jhh.2013.38

[28] Jones DW, Ferdinand KC, Taler SJ, Johnson HM, Shimbo D, Abdalla M, et al. AHA/ACC/AANP/AAPA/ABC/ACCP/ACPM/AGS/AMA/ASPC/NMA/PCNA/SGIM Guideline for the Prevention, Detection, Evaluation and Management of High Blood Pressure in Adults: A Report of the American College of Cardiology/American Heart Association Joint Committee on Clinical Practice Guidelines. Hypertension. 2025;82:e212–e316. 40811516 10.1161/HYP.0000000000000249

[29] Myers MG, Kaczorowski J. Office blood pressure is lower than awake ambulatory blood pressure at lower targets for treatment. J Clin Hypertens (Greenwich). 2017;19:1210–3. 28942618 10.1111/jch.13090 PMC8031269

[30] Mancia G, Parati G, Bilo G, Gao P, Fagard R, Redon J, et al. Ambulatory blood pressure values in the Ongoing Telmisartan Alone and in Combination with Ramipril Global Endpoint Trial (ONTARGET). Hypertension. 2012;60:1400–6. 23071122 10.1161/HYPERTENSIONAHA.112.199562

[31] Mancia G, Sega R, Bravi C, De Vito G, Valagussa F, Cesana G, et al. Ambulatory blood pressure normality: results from the PAMELA study. J Hypertens. 1995;13:1377–90. 8866899

[32] Myers MG, Matangi M, Kaczorowski J. Comparison of awake ambulatory blood pressure and automated office blood pressure using linear regression analysis in untreated patients in routine clinical practice. J Clin Hypertens (Greenwich). 2018;20:1696–702. 30328275 10.1111/jch.13409 PMC8030846

[33] Den Hond E, Celis H, Vandenhoven G, O’Brien E, Staessen JA, THOP investigators. Determinants of white-coat syndrome assessed by ambulatory blood pressure or self-measured home blood pressure. Blood Press Monit. 2003;8:37–40. 12604935 10.1097/00126097-200302000-00008

[34] Triantafyllidi H, Voutsinos D, Sioula K, Schoinas A, Benas D, Birmpa D, et al. Are unattended blood pressure measurements necessary in all patients visiting an outpatient cardiology clinic? High Blood Press Cardiovasc Prev. 2020;27:389–97. 32720295 10.1007/s40292-020-00402-0

