# Supplementary material for: Comparison of unattended, attended and routine clinic systolic blood pressure measurements and determinants of blood pressure difference between attended and unattended BP
Source: J Hum Hypertens. 2026 May 27;40(7):569–75. doi: 10.1038/s41371-026-01162-5 (PMC13345920; doi:10.1038/s41371-026-01162-5)
Supplement: Supplementary file 1 — Supplementary Table 1 [file 41371_2026_1162_MOESM1_ESM.docx]

|  | | **Mean BP (SD)** | **Mean Difference (SD)** | **P Value** | |
| --- | --- | --- | --- | --- | --- |
| **Difference between sequences stratified by </≥ 130 attended SBP** | **<130 attended SBP (n = 38)** | | | |  |
|  | Attended BP first (n = 12) | | | | |
|  | Attended | 117.67 (10.08) | 3.25 (8.19) | 0.197 | |
|  | Unattended | 114.42 (12.13) |  |  |  |
|  | Unattended BP first (n = 26) | | | | |
|  | Attended | 117.50 (9.74) | 0.88 (6.49) | 0.493 | |
|  | Unattended | 116.62 (10.57) |  |  |  |
|  | **≥ 130 attended SBP (n = 47)** | | | |  |
|  | Attended BP first (n = 31) | | | | |
|  | Attended | 150.61 (16.86) | 7.48 (6.67) | <0.001 | |
|  | Unattended | 143.13 (15.96) |  |  |  |
|  | Unattended BP first (n = 16) | | | | |
|  | Attended | 148.50 (12.21) | 3.50 (2.49) | 0.181 | |
|  | Unattended | 145.0 (16.45) |  |  |  |
|  | | | | | |
| **Difference between sequences stratified by </≥ 130 clinic SBP** | **<130 clinic SBP (n = 34)** | | | | |
|  | Attended BP first (n = 12) | | | | |
|  | Attended | 125.00 (16.11) | 4.58 (7.57) | 0.060 | |
|  | Unattended | 120.42 (18.37) |  |  |  |
|  | Unattended BP first (n = 22) | | | | |
|  | Attended | 117.50 (11.64) | 1.68 (6.66) | 0.250 | |
|  | Unattended | 115.82 (12.17) |  |  |  |
|  | **≥ 130 clinic SBP (n = 44)** | | | | |
|  | Attended BP first (n = 26) | | | | |
|  | Attended | 147.92 (19.62) | 6.85 (7.79) | <0.001 | |
|  | Unattended | 141.08 (17.03) |  |  |  |
|  | Unattended BP first (n = 18) | | | | |
|  | Attended | 140.44 (14.88) | 2.11 (9.44) | 0.356 | |
|  | Unattended | 138.33 (14.78) |  |  |  |

**Supplementary Table 1: Effect of sequence of blood pressure pre-measurement on mean difference in systolic blood pressure (SBP, in mmHg), when stratified by attended or routine clinic BP (<130 Vs. >130mmHg).**

*Supplementary Table 1 Abbreviations: SBP; systolic blood pressure, SD; standard deviation*
